# (±)-Evodiakine, A Pair of Rearranged Rutaecarpine-Type Alkaloids From *Evodia rutaecarpa*

**DOI:** 10.1007/s13659-016-0113-7

**Published:** 2016-11-21

**Authors:** Yan-Hong Li, Yu Zhang, Li-Yan Peng, Xiao-Nian Li, Qin-Shi Zhao, Rong-Tao Li, Xing-De Wu

**Affiliations:** 1 State Key Laboratory of Phytochemistry and Plant Resources in West China, Kunming Institute of Botany, Chinese Academy of Sciences, Kunming, 650201 People’s Republic of China; 2 Key Laboratory of Chemistry in Ethnic Medicinal Resources, State Ethnic Affairs Commission & Ministry of Education, School of Ethnic Medicine, Yunnan Minzu University, Jingming South Road, Chenggong New District, Kunming, 650504 Yunnan People’s Republic of China; 3 Faculty of Life Science and Technology, Kunming University of Science and Technology, Kunming, 650050 People’s Republic of China

**Keywords:** *Evodia rutaecarpa*, (±)-Evodiakine, Rutaecarpine-type alkaloids

## Abstract

**Abstract:**

(±)-Evodiakine (**1a** and **1b**), a pair of rearranged rutaecarpine-type alkaloids with an unprecedented 6/5/5/7/6 ring system, were isolated from the nearly ripe fruits of *Evodia rutaecarpa*. Separation of the enantiomers have been achieved by chiral HPLC column. The structures of (±)-evodiakine were unambiguously elucidated by 1D and 2D NMR spectra, mass spectrometry, and single-crystal X-ray diffraction. Their absolute configurations were determined by comparison of experimental and calculated electronic circular dichroism spectra. A hypothetical biogenetic pathway for (±)-evodiakine was also proposed. Compounds **1a**, **1b**, and the racemate (**1**) were tested for their cytotoxic and anti-inflammatory activities.

**Graphical Abstract:**

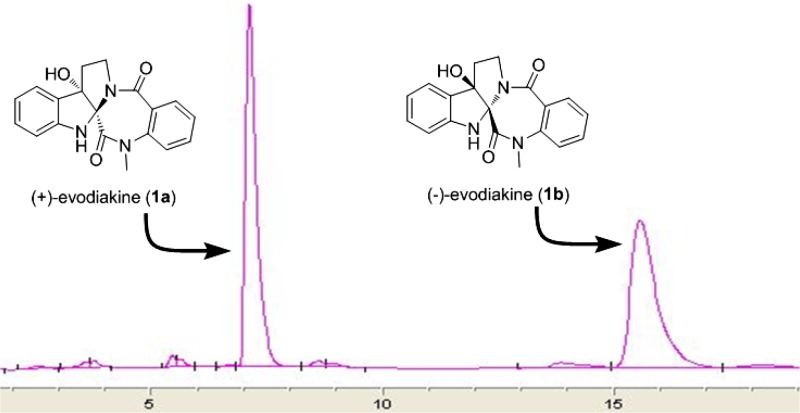

**Electronic supplementary material:**

The online version of this article (doi:10.1007/s13659-016-0113-7) contains supplementary material, which is available to authorized users.

## Introduction

**Fig. 1:**
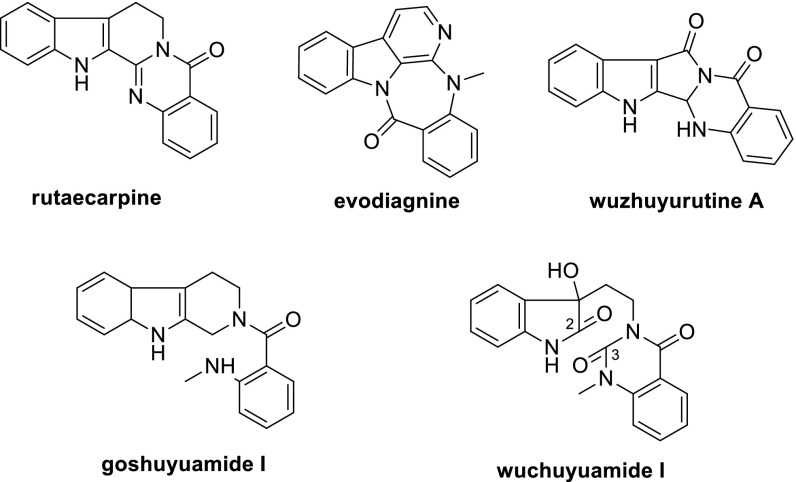
The known characteristic compounds from *E. rutaecarpa*

**Fig. 2:**
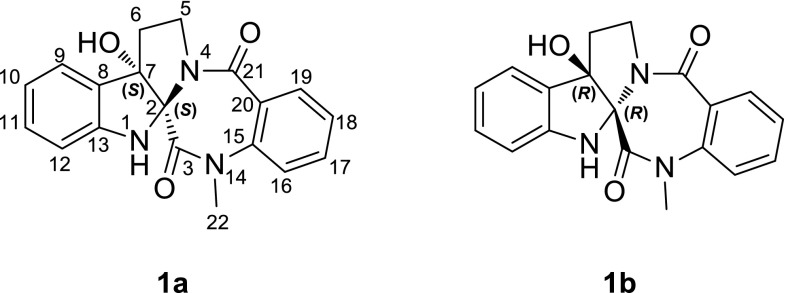
Structures of (+)-evodiakine (**1a**) and (−)-evodiakine (**1b**)

*Evodia rutaecarpa* (Juss.) Benth., a small tree belonging to the family Rutaceae mainly, is distributed in the south regions of Qinling in China [1]. The dried and nearly ripe fruits (Evodiae Fructus), called Wu-Zhu-Yu in Chinese (Goshuyu in Japanese), are used in traditional Chinese medicine for the treatment of various diseases, especially for treating headache, abdominal pain, migraines, chill limbs, postpartum hemorrhage, dysmenorrheal, diarrhea, nausea and hypertension [2]. Previous phytochemical study on *E. rutaecarpa* have focused on rutaecarpine-type alkaloids that produce a number of characteristic compounds with different ring system, including rutaecarpine (6/5/6/6/6) [3], evodiagnine (6/5/6/7/6) [4], and wuzhuyurutine A (6/5/5/6/6) [5], as well as several *seco*-derivatives, such as goshuyuamide I [6] and wuchuyuamide I [7] (Fig. 1). Our previous study on this plant reported three new alkaloids, evollionines A–C, together with evollionines A and B belonging to *seco*-ring rutaecarpine-type alkaloids [8]. A continuous study on the plant led to the isolation of a pair of rearranged rutaecarpine-type derivatives, (+)-evodiakine (**1a**) and (−)-evodiakine (**1b**), containing the interesting 6/5/5/7/6 ring system depicted as in Fig. 2. Their structure elucidation was unambiguously achieved by spectroscopic data, single-crystal X-ray diffraction, and electronic circular dichroism (ECD).

## Results and Discussion

Evodiakine (**1**) was obtained as colorless needles. Its molecular formula C_19_H_17_N_3_O_3_ was deduced from the HR-EI-MS at *m/z* 335.1284 ([M]^+^, calc. 335.1270), corresponding to 13 degrees of unsaturation. The characteristic absorption at 3409 (OH and/or NH), 1602 and 1467 cm^**−**1^ (aromatic ring) in IR spectrum, and at 203, 218, 288 nm in UV spectrum, together with a OH signal (*δ*_H_ 6.49, s) and a NH (*δ*_H_ 6.00, s) signal in the ^1^H NMR spectrum (Table 1) implied the possibility of a dihydroindoline derivative for **1** [7]. In addition, the proton signals at *δ*_H_ 6.56 (d, *J* = 7.7 Hz), 7.08 (t, *J* = 7.7 Hz), 6.81 (t, *J* = 7.7 Hz), 7.33 (d, *J* = 7.7 Hz); and *δ*_H_ 7.58 (d, *J* = 8.0 Hz), 7.74 (t, *J* = 8.0 Hz), 7.43 (t, *J* = 8.0 Hz), 7.72 (d, *J* = 8.0 Hz) displayed characteristic of two ortho-disubstituted benzenoid rings. The ^13^C NMR, DEPT, and HSQC spectra suggested that **1** exhibited seven other carbon signals due to two aliphatic methylenes, four quaternary carbons (two amide groups at *δ*_C_ 164.3 and 168.6, one OH-substituted quaternary carbon at *δ*_C_ 79.0, and one carbinolamine quaternary carbon at *δ*_C_ 91.2), and one *N*-methyl (*δ*_H_ 3.43; *δ*_C_ 36.4). These spectroscopic data suggested that **1** was an alkaloid with polyheterocyclic systems.

**Table 1:** NMR data for compound **1** in DMSO-*d*_6_. (600 MHz for ^1^H and 150 MHz for ^13^C)

Position	*δ*_H_, mult. (*J* in Hz)	*δ*_C_, mult.	HMBC (^1^H–^13^C)
2		91.2, C	
3		168.6, C	
5	3.87, m 3.10, m	45.8, CH_2_	2, 6, 7
6	2.65, m 2.35, m	33.1, CH_2_	2, 5, 7, 8
7		79.0, C	
8		130.1, C	
9	6.56, d (7.7)	111.9, CH	8, 11
10	7.08, t (7.7)	129.8, CH	12, 13
11	6.81, t (7.7)	119.9, CH	9, 10
12	7.33, d (7.7)	123.2, CH	2, 10, 13
13		148.2, C	
15		138.3, C	
16	7.58, d (8.0)	123.3, CH	15, 17, 20, 21
17	7.74, t (8.0)	132.9, CH	15, 16, 19
18	7.43, t (8.0)	126.2, CH	15, 16, 20
19	7.72, d (8.0)	130.2, CH	15, 17, 21
20		127.9, C	
21		164.3, C	
N-Me	3.43, s	36.4, CH_3_	3, 15
N-H	6.00, s		2, 3, 7, 8, 13
7-OH	6.49, s		2, 7, 8

**Fig. 3:**
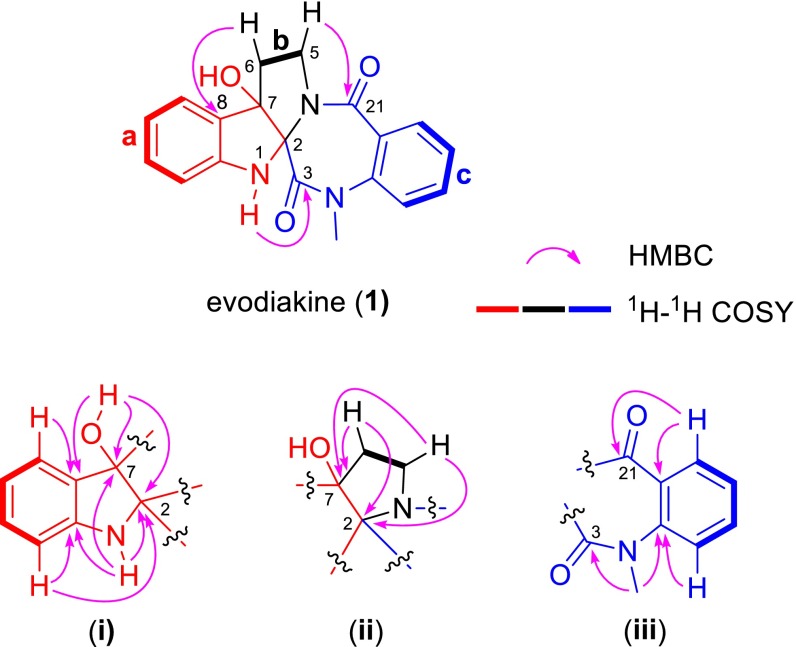
Key HMBC and COSY correlations of evodiakine (**1**)

**Fig. 4:**
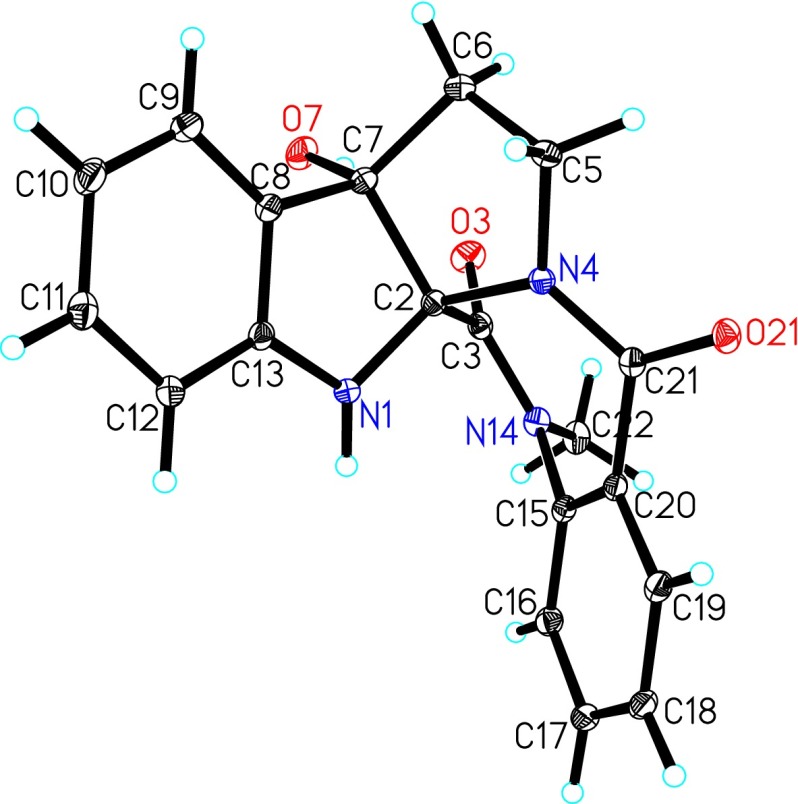
X-ray crystal structure of evodiakine (**1**)

The structure of **1** was established by the further 2D NMR data analysis. In the ^1^H-^1^H COSY spectrum (Fig. 3), the cross peaks of H-9/H-10/H-11/H-12 and H-16/H-17/H-18/H-19 confirmed the presence of the two H-bearing structural fragments (**a** and **c**) and consistent with the ^1^H NMR spectrum. Additionally, the ^1^H-^1^H COSY correlations of H_2_-5/H_2_-6 suggested the existence of a CH_2_CH_2_ alkyl fragment (**b**). The fragment of **a** together with the HMBC correlations from OH-7 (*δ*_H_ 6.49) to C-2, C-7, and C-8, from N_1_-H (*δ*_H_ 6.00) to C-2, C-7, C-8, and C-13, from H-9 with C-8 and C-11, and from H-12 to C-2, C-10, and C-13 revealed the presence of a 2,3-disubstituted indolin-3-ol moiety (**i**) in **1**. The HMBC correlations of H_2_-5 with C-2, C-6, and C-7 and of H_2_-6 with C-2, C-5, C-7, and C-8 demonstrated that the indolin-3-ol moiety (**i**) was fused with a pyrrolidine ring (**ii**) via C-2 and C-7. In addition, the rest structural fragment (**iii**) was established by the HMBC correlations of H-16 with C-15, C-17, C-20, and C-21, of H-19 with C-15, C-17, and C-21, and of H_3_-22 with C-3 and C-15. Furthermore, the HMBC correlation of N_1_-H with C-3, as well as the downfield chemical shift of C-2 at *δ*_C_ 91.2, suggested that the carbonyl group (C-3) was connected to the C-2 of the indolin-3-ol moiety (**i**). Finally, the absence of an N-4 proton resonance in the ^1^H NMR spectrum combined with the molecular composition suggested that C-21 was linked to N-4 of the pyrrolidine moiety (**ii**). Thus, the structure of **1** was identified as a pentacyclic alkaloid by fusing indole and the *seco*-pyrroloquinazolone rings [9] (Fig. 3). This deduction was further confirmed by an X-ray diffraction experiment using Mo Kα radiation (Fig. 4).

**Fig. 5:**
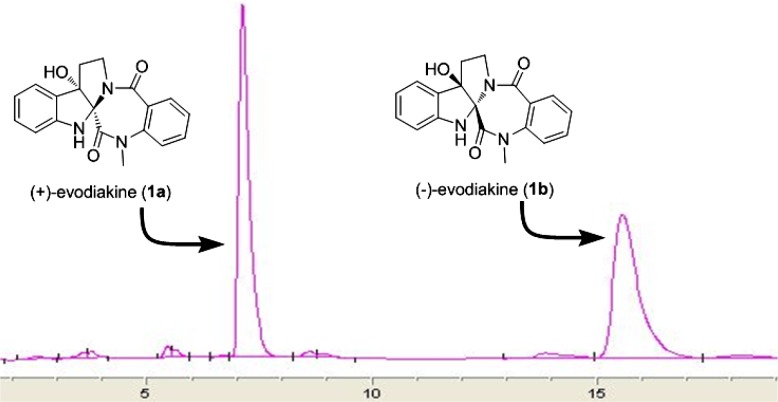
The HPLC profiles of separation of (**+**)- and (−)-evodiakine on chiral IC column

**Fig. 6:**
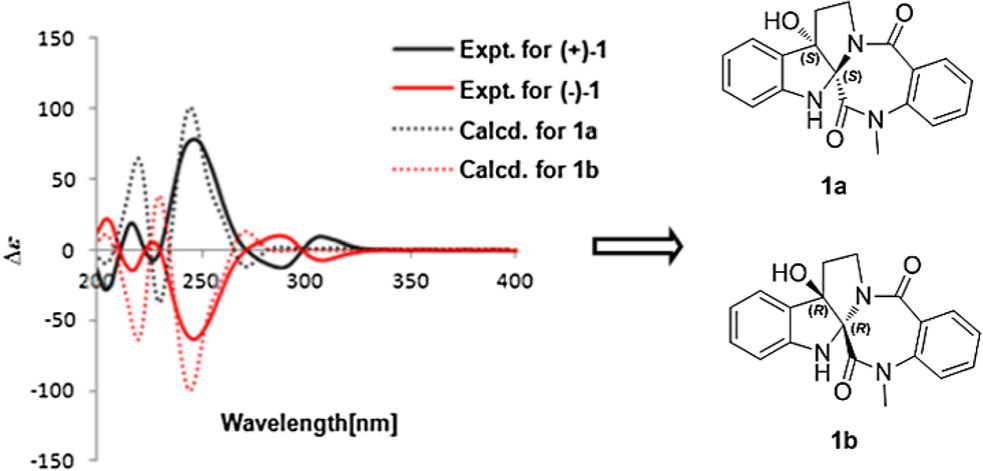
Experimental and calculated ECD spectra of compounds **1a** and **1b**

Evodiakine (**1**) was optically inactive, [α]_D_^24.8^ ≈ 0 (c 0.12, MeOH), indicating that it was obtained as a racemate. Subsequent HPLC separation of **1** on a chiral column prior to HPLC separation yielded two compounds, **1a** and **1b** (Fig. 5). However, the isolated compounds showed opposite optical rotation, and their ECD spectra displayed mirror curves as shown in Fig. 6. This confirmed the successful separation of enantiomers, (**+**)-evodiakine (**1a**) and (−)-evodiakine (**1b**). To secure unambiguous confirmation of the absolute configuration of compounds **1a** and **1b**, the ECD calculation at the B3LYP/6-31G** level in Gaussian 03 program package was carried out, which provided vindication of their configuration [10]. In the 200–400 nm region, the calculated ECD spectra of compounds **1a** and **1b** were consistent with the experimental ECD spectra of (**+**)-**1** and (−)-**1**, respectively. Thus, the absolute configuration of compound **1a** was determined to be 2*S*,7*S*-evodiakine, as well as that of **1b** was revealed as 2*R*,7*R*-evodiakine.

**Scheme 1:**
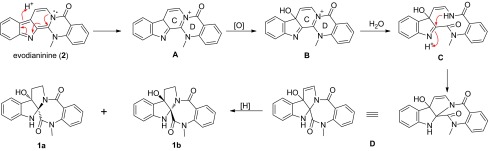
Plausible biogenetic pathway of (+)-**1a** and (−)-**1b**

By comparison with known rutaecarpine-type alkaloids, compound (±)-**1** was regarded as a rearranged rutaecarpine-type alkaloid with an unprecedented 6/5/5/7/6 ring system. From a biogenetic point of view, (±)-evodialine (**1**) would plausibly be derived from a common rutaecarpine-type alkaloid, evodianinine (**2**), via the sequence shown in Scheme 1. Compound **2** underwent isomerization and oxidation to produce intermediate **B** [11]. Then, the C-3/N-4 bond cleavage and the formation of a heptatomic ring accomplished via the key intermediate **C** [12]. The rings C and D rearrangement of **B** via attack of the NH in **C** onto the imine carbon yielded the structure **D**, which was followed by reduction of the C-5/C-6 double bond to produce compounds **1a** and **1b** [13, 14].

Compounds **1a**, **1b**, and the racemate (**1**) were evaluated for their anti-inflammatory and cytotoxic activities, but none of them showed ability to inhibit NO production of LPS-stimulated RAW 264.7 macrophages, as well as cytotoxicity against HL-60, SMMC-7721, A-549, MCF-7, and SW-480 cancer cell lines with IC_50_ values of more than 40 μM.

## Experimental Section

### General

Melting points were obtained on an X-4 micro melting point apparatus. Optical rotations were measured with a Perkin-Elmer model 241 polarimeter. UV spectra were recorded using a Shimadzu UV-2401A spectrophotometer. IR spectra were determined on a Tensor-27 infrared spectrophotometer with KBr pellets. ECD spectra were obtained on a JASCO J-810 spectrophotometer. 1D and 2D NMR spectra were performed on Avance III-600 spectrometers with TMS as an internal standard. ESIMS and HREIMS were measured using an API-QSTAR-Pulsar-1 or VG Auto Spec-3000 instruments. X-ray diffraction was conducted using Bruker APEX DUO diffractometer with graphite-monochromated MoKα radiation. MPLC was performed on a Lisui EZ Purify III System including pump manager P03, detector modules P02, and fraction collector P01 (Shanghai Lisui Chemical Engineering Co., Ltd., Shanghai, China). Column chromatography (CC) was performed over silica gel (200–300 mesh, Qingdao Marine Chemical Co. Ltd., Qingdao, China), MCI gel (CHP 20P, 75–150 μm, Mitsubishi Chemical Corporation, Japan). Thin-layer chromatography (TLC) was carried out on silica gel GF_254_ on glass plates (Qingdao Marine Chemical Co. Ltd.) using various solvent systems and spots were visualized by spraying improved Dragendorff’s reagent to the silica gel plates.

### Plant Materials

The dried and nearly ripe fruits of *E. rutaecarpa* were purchased from the Kunming Ju-Hua village pharmaceutical sale market, Yunnan province, P. R. China.

### Extraction and Isolation

The dried and near ripe fruits of *E. rutaecarpa* (20.0 kg) were extracted with MeOH (3 × 10 L) at room temperature for 24 h each time. The MeOH extracts were evaporated under reduced pressure to give a residue, which was dissolved in 1% HCl, acidified to pH 2, and then partitioned with EtOAc (3 × 4 L). The acidic solution was basified using saturated Na_2_CO_3_ to pH 10, followed by exhaustive extraction with CHCl_3_ (3 × 4 L) to afford a extract (36 g). The CHCl_3_ extract was subjected to MPLC (MCI, 500.0 g, 25 × 4 cm) eluted with MeOH/H_2_O (40 → 100%) to yield four fractions (Frs. I–IV). Fr. III (10 g) was applied to repeated CC (SiO_2_, 250.0 g, 50 × 3 cm, CHCl_3_/acetone 9:1; SiO_2_, 350.0 g, 60 × 5 cm, CH_3_Cl/MeOH/Et_2_NH 49:1:0.3) to provide compound **1** (13.0 mg).

#### Evodiakine (**1**)

Colorless needles (cyclohexane/acetone, 2:1); mp 218 ~ 220 °C; [α]_D_^24.8^ ≈ 0 (c 0.12, MeOH); UV (MeOH) *λ*_max_ (log ε): 288 (3.43), 218 (4.35), 203 (4.38) nm; IR (KBr) *ν*_max_: 3409, 2923, 1637, 1602, 1467 cm^−1^; ^1^H and ^13^C NMR data, see Table 1; ESIMS *m*/*z* 358 [M + Na]^+^; HREIMS *m*/*z* 335.1284 ([M]^+^, calcd for C_19_H_17_N_3_O_3_: 335.1270). (**+**)-evodiakine (**1a**): [α]_D_^25.3^ + 421.1 (c 0.15, MeOH); ECD (MeOH) *λ*_max_ (Δ*ε*): 204 (−19.45), 216 (+12.97), 227 (−5.12), 246 (+53.85), 287 (−8.65), 306 (+6.38) nm; (−**)**-evodiakine (**1b**): [α]_D_^25.3^ −422.7 (c 0.20, MeOH); ECD (MeOH) *λ*_max_ (Δ*ε*): 205 (+14.61), 217 (−9.81), 227 (+3.61), 246 (−42.96), 287 (+6.60), 308 (−5.02) nm.

#### Crystal Data for Evodiakine (**1**)

C_19_H_17_N_3_O_3_·C_6_H_12_, *M* = 419.51, monoclinic, *a* = 19.629(2) Å, *b* = 10.1354(13) Å, *c* = 10.7440(13) Å, *α* = 90.00°, *β* = 98.410(2)°, *γ* = 90.00°, *V* = 2114.5(5) Å^3^, *T* = 100(2) K, space group *P*21*/c*, *Z* = 4, *μ*(MoKα) = 0.087 mm^−1^, 21663 reflections measured, 5933 independent reflections (*R*_*int*_ = 0.0312). The final *R*_1_ values were 0.0516 (*I* > 2*σ*(*I*)). The final *wR*(*F* ^2^) values were 0.1530 (*I* > 2*σ*(*I*)). The final *R*_1_ values were 0.0655 (all data). The final *wR*(*F* ^2^) values were 0.1681 (all data). The goodness of fit on *F* ^2^ was 1.014. Crystallographic data for **1** reported in this paper have been deposited at the Cambridge Crystallographic Data Centre, under reference number CCDC 1486886.

### Inhibition of Nitric Oxide Production Assay

The inhibitory effects of the test compounds on NO production were evaluated based on a detection model with LPS-activated murine macrophage RAW264.7 cells, which was performed as described previously [15]. The concentration of NO in the cultured medium was measured indirectly by analysis of nitrite levels using the Griess reaction. MG-132 was used as a positive control.

### Cytotoxicity Assay

The isolates were tested in vitro for their cytotoxicities to inhibit proliferation of five human tumour cell lines, HL-60, SMMC-7721, A-549, MCF-7, and SW480. Cell viability was assessed by conducting colorimetric measurements of the amount of insoluble formazan formed in mitochondrion of living cells according to the MTT method [16]. In brief, each cancer cell line was exposed to the compounds dissolved in DMSO at five different concentrations in triplicate for 48 h, with *cis*-platin as a positive control.

## Electronic supplementary material

Below is the link to the electronic supplementary material.

Supplementary material 1 (DOCX 704 kb)
