# Supplementary material for: (±)-Evodiakine, A Pair of Rearranged Rutaecarpine-Type Alkaloids From Evodia rutaecarpa
Source: Nat Prod Bioprospect. 2016 Nov 21;6(6):291–6. doi: 10.1007/s13659-016-0113-7 (PMC5136374; doi:10.1007/s13659-016-0113-7)
Supplement: Supplementary file 1 — Supplementary material 1 (DOCX 704 kb) [file 13659_2016_113_MOESM1_ESM.docx]

**Supplementary data**

(±)-Evodiakine, A Pair of Rearranged Rutaecarpine-type Alkaloids from *Evodia rutaecarpa*

Yan-Hong Li Yu Zhang Li-Yan Peng Xiao-Nian Li Qin-Shi Zhao Rong-Tao Li Xing-De Wu

Y.-H. Li Y. Zhang L.-Y. Peng X.-N. Li Q.-S. Zhao X.-D. Wu (🖂)

State Key Laboratory of Phytochemistry and Plant Resources in West China, Kunming Institute of Botany, Chinese Academy of Sciences, Kunming 650201, People’s Republic of China.

e-mail: wuxingde@mail.kib.ac.cn

Y.-H. Li

Key Laboratory of Chemistry in Ethnic Medicinal Resources, State Ethnic Affairs Commission & Ministry of Education, School of Ethnic Medicine, Yunnan Minzu University, Jingming South Road, Chenggong New District, Kunming, Yunnan, 650504, P. R. China.

Y.-H. Li R.-T. Li (🖂)

Faculty of Life Science and Technology, Kunming University of Science and Technology, Kunming 650050, PR China.

e-mail: lrt512@163.com **Notes**

| **Figure S1**. IR spectrum of (**±**)-evodiakine (**1**). | 1 |
| --- | --- |
| **Figure S2**. HREIMS spectrum of (**±**)-evodiakine (**1**). | 1 |
| **Figure S3**. ^1^H NMR spectrum (600 MHz, DMSO-*d*_6_) of (**±**)-evodiakine (**1**). | 2 |
| **Figure S4**. ^13^C NMR spectrum (150 MHz, DMSO-*d*_6_) of (**±**)-evodiakine (**1**). | 3 |
| **Figure S5**. HSQC spectrum (600 MHz, DMSO-*d*_6_) of (**±**)-evodiakine (**1**). | 4 |
| **Figure S6**. HMBC spectrum (600 MHz, DMSO-*d*_6_) of (**±**)-evodiakine (**1**). | 5 |
| **Figure S7**. COSY spectrum (600 MHz, DMSO-*d*_6_) of (**±**)-evodiakine (**1**). | 6 |
| **Figure S8**. ROESY spectrum (600 MHz, DMSO-*d*_6_) of (**±**)-evodiakine (**1**). | 7 |
| **Figure S9**. Crystal Cell Diagram for (**±**)- evodiakine (**1**). | 8 |
| **Figure S10**. Atomic Coordinates ($\times$10^4^) and Equivalent Isotropic Displacement Parameters for the Atoms of (**±**)-evodiakine (**1**). | 8 |
| **Figure S11**. Bond Lengths and Angles for (**±**)-evodiakine (**1**). | 9 |
| **Figure S12.** The HPLC profiles of separation of (+)-evodiakine (**1a**) and (-)-evodiakine **(1b**). | 12 |
| **Figure S13.** ECD spectra of (+)-evodiakine (**1a**) and (-)-evodiakine **(1b**). | 13 |

**Figure S1**. IR spectrum of (**±**)-evodiakine (**1**)

**Figure S2**. HREIMS spectrum of (**±**)-evodiakine (**1**)


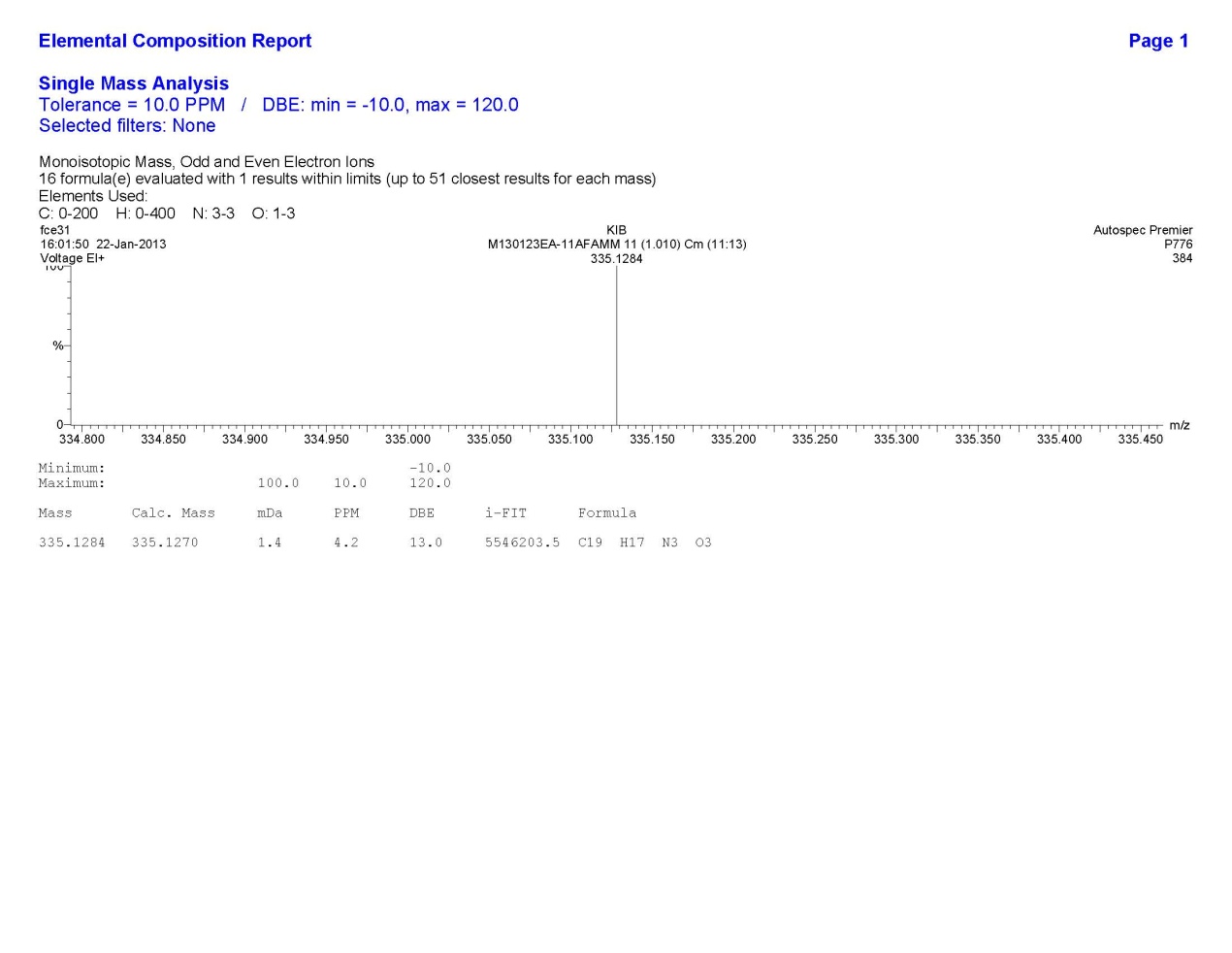


**Figure S3**. ^1^H NMR spectrum (600 MHz, DMSO-*d*_6_) of (**±**)-evodiakine (**1**)


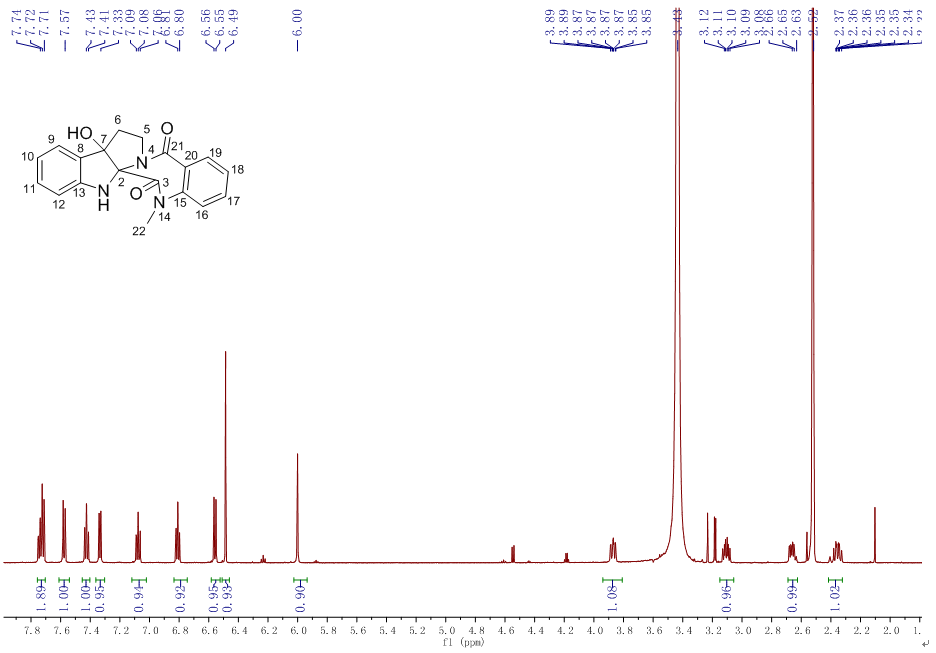


**Figure S4**. ^13^C NMR spectrum (150 MHz, DMSO-*d*_6_) of (**±**)-evodiakine (**1**)


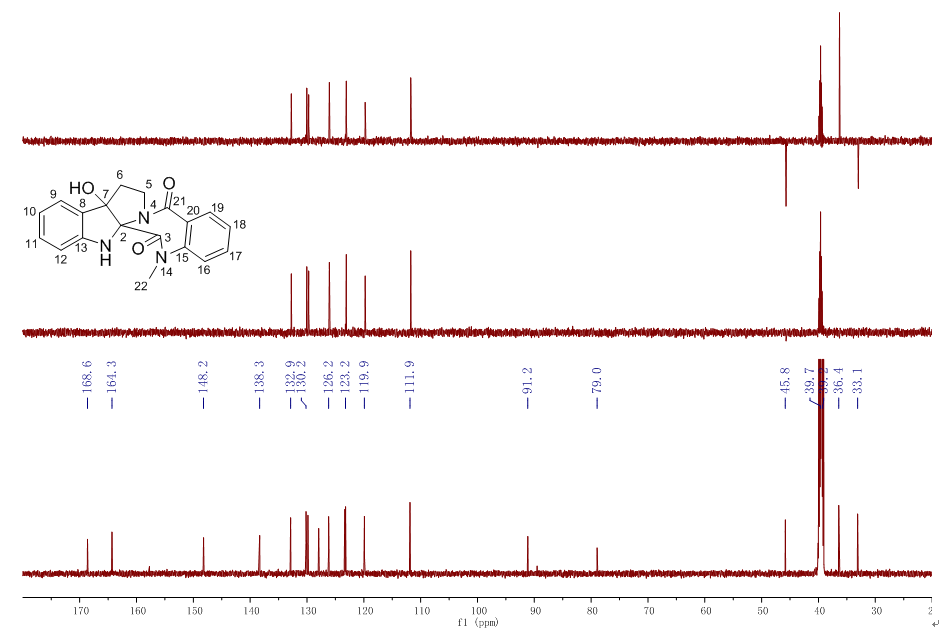


**Figure S5**. HSQC spectrum (600 MHz, DMSO-*d*_6_) of (**±**)-evodiakine (**1**)


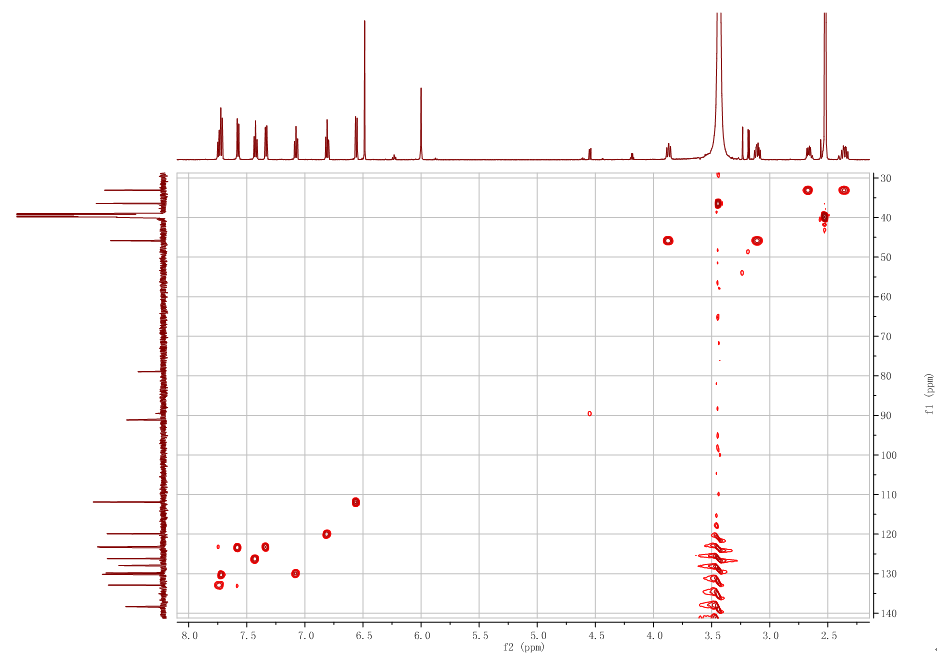


**Figure S6**. HMBC spectrum (600 MHz, DMSO-*d*_6_) of (**±**)-evodiakine (**1**)


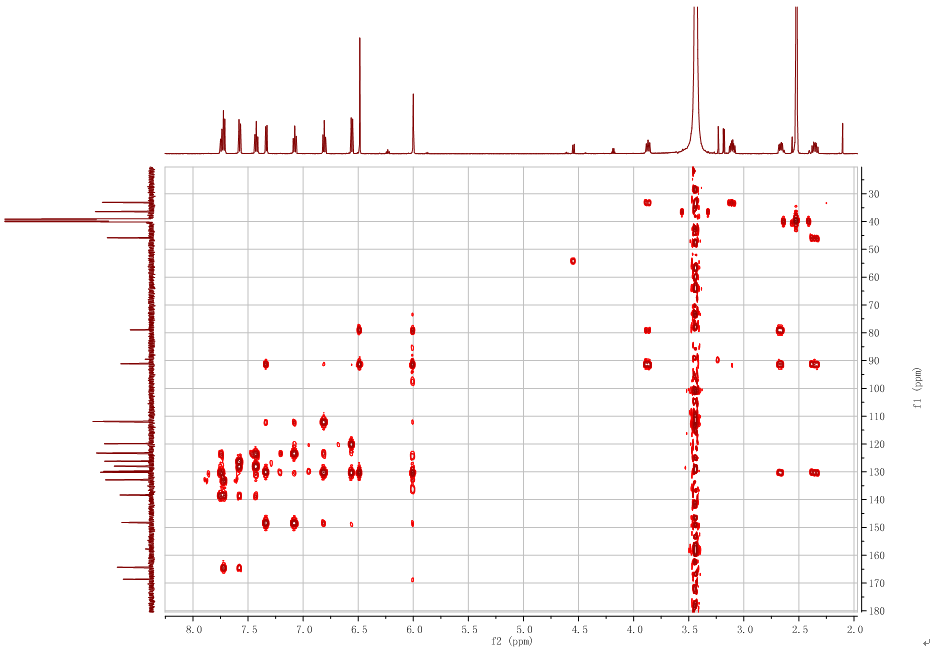


**Figure S7**. COSY spectrum (600 MHz, DMSO-*d*_6_) of (**±**)-evodiakine (**1**)


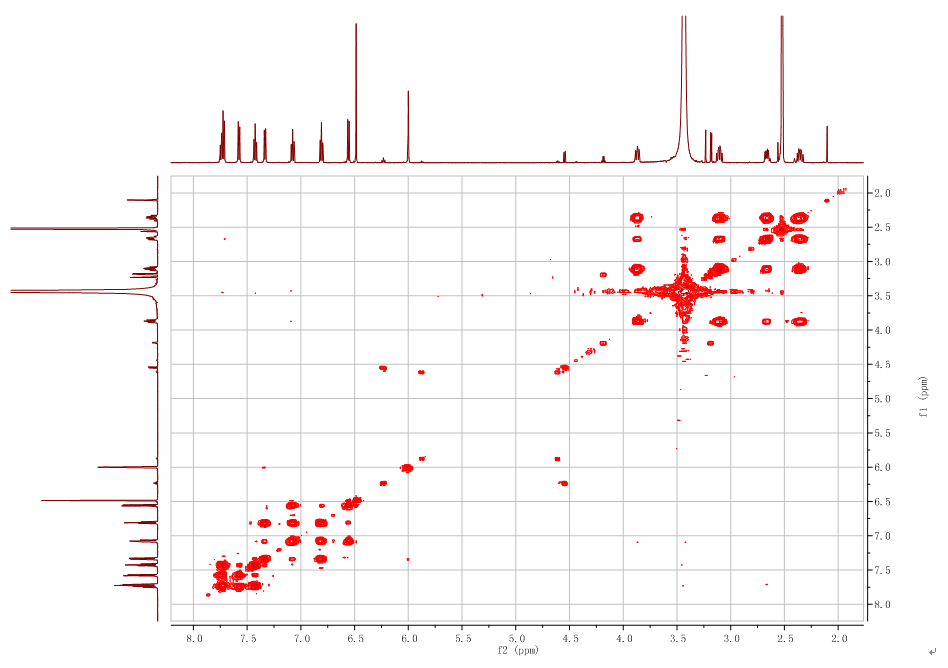


**Figure S8**. ROESY spectrum (600 MHz, DMSO-*d*_6_) of (**±**)-evodiakine (**1**)


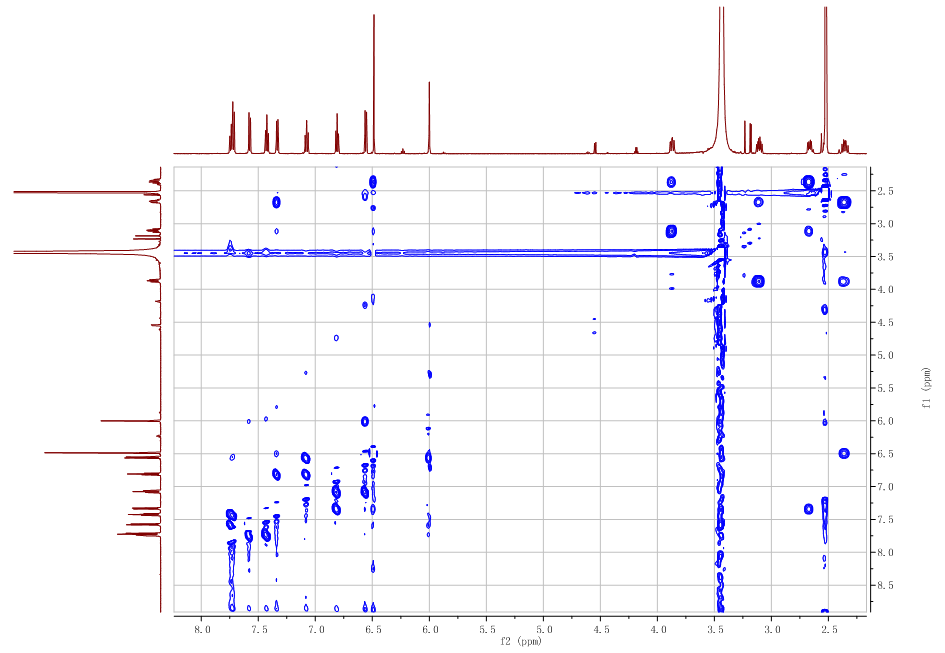


**Figure S9**. Crystal Cell Diagram for (**±**)-evodiakine (**1**).

**Figure S10**. Atomic Coordinates ($\times$10^4^) and Equivalent Isotropic Displacement Parameters for the Atoms of (**±**)-evodiakine (**1**).

________________________________________________________________

x y z U(eq)

________________________________________________________________

O(14) 666(1) 3629(1) 2255(1) 24(1)

O(5) 2079(1) 7608(1) 4038(1) 20(1)

O(8A) 1765(1) 2270(1) 1933(1) 24(1)

N(6) 1974(1) 5656(1) 3015(1) 15(1)

N(13) 1912(1) 5098(1) 737(1) 15(1)

N(15) 542(1) 5740(1) 1602(1) 17(1)

C(3) 1179(1) 9567(1) 756(1) 21(1)

C(4) 1541(1) 8891(1) 1764(1) 18(1)

C(4A) 1371(1) 7588(1) 2036(1) 15(1)

C(5) 1822(1) 6951(1) 3118(1) 15(1)

C(13A) 1712(1) 4774(1) 1950(1) 14(1)

C(12A) 2560(1) 4534(1) 684(1) 16(1)

C(12) 3017(1) 4802(1) -162(1) 18(1)

C(11) 3625(1) 4068(2) -41(1) 23(1)

C(2) 642(1) 8942(2) -8(1) 21(1)

C(15A) 806(1) 6990(1) 1296(1) 16(1)

C(16) -207(1) 5636(2) 1596(1) 21(1)

C(14) 931(1) 4658(1) 1935(1) 16(1)

C(7) 2464(1) 5000(2) 4001(1) 19(1)

C(8) 2306(1) 3541(1) 3778(1) 21(1)

C(8A) 2120(1) 3446(1) 2350(1) 18(1)

C(8B) 2714(1) 3577(1) 1620(1) 18(1)

C(9) 3316(1) 2853(2) 1728(1) 23(1)

C(10) 3778(1) 3107(2) 884(2) 25(1)

C(1) 452(1) 7669(1) 268(1) 19(1)

C(1S) 4636(1) 8119(2) 6389(2) 44(1)

C(2S) 4137(1) 8312(2) 7333(2) 41(1)

C(3S) 3517(1) 7409(2) 7067(2) 41(1)

C(4S) 3730(1) 5980(2) 6974(2) 37(1)

C(6S) 4842(1) 6685(2) 6290(2) 42(1)

C(5S) 4221(1) 5796(2) 6013(2) 40(1)

________________________________________________________________

**Figure S11**. Bond Lengths and Angles for (**±**)-evodiakine (**1**).

_____________________________________________________________

O(14)-C(14) 1.2364(17)

O(5)-C(5) 1.2369(16)

O(8A)-C(8A) 1.4204(17)

O(8A)-H(8A) 0.8400

N(6)-C(5) 1.3541(17)

N(6)-C(7) 1.4804(17)

N(6)-C(13A) 1.4834(16)

N(13)-C(12A) 1.4031(17)

N(13)-C(13A) 1.4536(16)

N(13)-H(13) 0.8800

N(15)-C(14) 1.3550(18)

N(15)-C(15A) 1.4253(17)

N(15)-C(16) 1.4739(17)

C(3)-C(4) 1.386(2)

C(3)-C(2) 1.390(2)

C(3)-H(3) 0.9500

C(4)-C(4A) 1.4033(19)

C(4)-H(4) 0.9500

C(4A)-C(15A) 1.4049(18)

C(4A)-C(5) 1.4993(18)

C(13A)-C(14) 1.5350(18)

C(13A)-C(8A) 1.5920(19)

C(12A)-C(12) 1.3935(19)

C(12A)-C(8B) 1.3976(19)

C(12)-C(11) 1.397(2)

C(12)-H(12) 0.9500

C(11)-C(10) 1.393(2)

C(11)-H(11) 0.9500

C(2)-C(1) 1.387(2)

C(2)-H(2) 0.9500

C(15A)-C(1) 1.3979(19)

C(16)-H(16A) 0.9800

C(16)-H(16B) 0.9800

C(16)-H(16C) 0.9800

C(7)-C(8) 1.522(2)

C(7)-H(7A) 0.9900

C(7)-H(7B) 0.9900

C(8)-C(8A) 1.5273(19)

C(8)-H(8B) 0.9900

C(8)-H(8C) 0.9900

C(8A)-C(8B) 1.5031(19)

C(8B)-C(9) 1.382(2)

C(9)-C(10) 1.396(2)

C(9)-H(9) 0.9500

C(10)-H(10) 0.9500

C(1)-H(1) 0.9500

C(1S)-C(6S) 1.516(3)

C(1S)-C(2S) 1.523(3)

C(1S)-H(1S1) 0.9900

C(1S)-H(1S2) 0.9900

C(2S)-C(3S) 1.516(3)

C(2S)-H(2S1) 0.9900

C(2S)-H(2S2) 0.9900

C(3S)-C(4S) 1.515(3)

C(3S)-H(3S1) 0.9900

C(3S)-H(3S2) 0.9900

C(4S)-C(5S) 1.523(3)

C(4S)-H(4S1) 0.9900

C(4S)-H(4S2) 0.9900

C(6S)-C(5S) 1.509(3)

C(6S)-H(6S1) 0.9900

C(6S)-H(6S2) 0.9900

C(5S)-H(5S1) 0.9900

C(5S)-H(5S2) 0.9900

C(8A)-O(8A)-H(8A) 109.5

C(5)-N(6)-C(7) 120.36(11)

C(5)-N(6)-C(13A) 126.24(11)

C(7)-N(6)-C(13A) 113.37(11)

C(12A)-N(13)-C(13A) 108.03(10)

C(12A)-N(13)-H(13) 126.0

C(13A)-N(13)-H(13) 126.0

C(14)-N(15)-C(15A) 124.78(11)

C(14)-N(15)-C(16) 117.88(12)

C(15A)-N(15)-C(16) 117.31(11)

C(4)-C(3)-C(2) 119.58(13)

C(4)-C(3)-H(3) 120.2

C(2)-C(3)-H(3) 120.2

C(3)-C(4)-C(4A) 121.20(13)

C(3)-C(4)-H(4) 119.4

C(4A)-C(4)-H(4) 119.4

C(4)-C(4A)-C(15A) 118.69(12)

C(4)-C(4A)-C(5) 115.86(12)

C(15A)-C(4A)-C(5) 125.45(12)

O(5)-C(5)-N(6) 121.18(12)

O(5)-C(5)-C(4A) 120.85(12)

N(6)-C(5)-C(4A) 117.81(11)

N(13)-C(13A)-N(6) 116.53(11)

N(13)-C(13A)-C(14) 114.10(11)

N(6)-C(13A)-C(14) 106.74(10)

N(13)-C(13A)-C(8A) 103.98(10)

N(6)-C(13A)-C(8A) 101.41(10)

C(14)-C(13A)-C(8A) 113.55(11)

C(12)-C(12A)-C(8B) 120.87(12)

C(12)-C(12A)-N(13) 128.30(12)

C(8B)-C(12A)-N(13) 110.81(12)

C(12A)-C(12)-C(11) 117.25(13)

C(12A)-C(12)-H(12) 121.4

C(11)-C(12)-H(12) 121.4

C(10)-C(11)-C(12) 122.04(14)

C(10)-C(11)-H(11) 119.0

C(12)-C(11)-H(11) 119.0

C(1)-C(2)-C(3) 120.17(13)

C(1)-C(2)-H(2) 119.9

C(3)-C(2)-H(2) 119.9

C(1)-C(15A)-C(4A) 119.70(13)

C(1)-C(15A)-N(15) 117.65(12)

C(4A)-C(15A)-N(15) 122.50(12)

N(15)-C(16)-H(16A) 109.5

N(15)-C(16)-H(16B) 109.5

H(16A)-C(16)-H(16B) 109.5

N(15)-C(16)-H(16C) 109.5

H(16A)-C(16)-H(16C) 109.5

H(16B)-C(16)-H(16C) 109.5

O(14)-C(14)-N(15) 120.82(13)

O(14)-C(14)-C(13A) 121.46(13)

N(15)-C(14)-C(13A) 117.67(12)

N(6)-C(7)-C(8) 103.16(11)

N(6)-C(7)-H(7A) 111.1

C(8)-C(7)-H(7A) 111.1

N(6)-C(7)-H(7B) 111.1

C(8)-C(7)-H(7B) 111.1

H(7A)-C(7)-H(7B) 109.1

C(7)-C(8)-C(8A) 103.48(11)

C(7)-C(8)-H(8B) 111.1

C(8A)-C(8)-H(8B) 111.1

C(7)-C(8)-H(8C) 111.1

C(8A)-C(8)-H(8C) 111.1

H(8B)-C(8)-H(8C) 109.0

O(8A)-C(8A)-C(8B) 106.96(11)

O(8A)-C(8A)-C(8) 113.82(12)

C(8B)-C(8A)-C(8) 115.37(12)

O(8A)-C(8A)-C(13A) 114.88(11)

C(8B)-C(8A)-C(13A) 100.53(11)

C(8)-C(8A)-C(13A) 104.75(11)

C(9)-C(8B)-C(12A) 121.38(13)

C(9)-C(8B)-C(8A) 128.96(13)

C(12A)-C(8B)-C(8A) 109.53(12)

C(8B)-C(9)-C(10) 118.43(14)

C(8B)-C(9)-H(9) 120.8

C(10)-C(9)-H(9) 120.8

C(11)-C(10)-C(9) 120.02(13)

C(11)-C(10)-H(10) 120.0

C(9)-C(10)-H(10) 120.0

C(2)-C(1)-C(15A) 120.55(13)

C(2)-C(1)-H(1) 119.7

C(15A)-C(1)-H(1) 119.7

C(6S)-C(1S)-C(2S) 111.94(17)

C(6S)-C(1S)-H(1S1) 109.2

C(2S)-C(1S)-H(1S1) 109.2

C(6S)-C(1S)-H(1S2) 109.2

C(2S)-C(1S)-H(1S2) 109.2

H(1S1)-C(1S)-H(1S2) 107.9

C(3S)-C(2S)-C(1S) 111.92(18)

C(3S)-C(2S)-H(2S1) 109.2

C(1S)-C(2S)-H(2S1) 109.2

C(3S)-C(2S)-H(2S2) 109.2

C(1S)-C(2S)-H(2S2) 109.2

H(2S1)-C(2S)-H(2S2) 107.9

C(4S)-C(3S)-C(2S) 111.57(16)

C(4S)-C(3S)-H(3S1) 109.3

C(2S)-C(3S)-H(3S1) 109.3

C(4S)-C(3S)-H(3S2) 109.3

C(2S)-C(3S)-H(3S2) 109.3

H(3S1)-C(3S)-H(3S2) 108.0

C(3S)-C(4S)-C(5S) 111.74(16)

C(3S)-C(4S)-H(4S1) 109.3

C(5S)-C(4S)-H(4S1) 109.3

C(3S)-C(4S)-H(4S2) 109.3

C(5S)-C(4S)-H(4S2) 109.3

H(4S1)-C(4S)-H(4S2) 107.9

C(5S)-C(6S)-C(1S) 111.81(17)

C(5S)-C(6S)-H(6S1) 109.3

C(1S)-C(6S)-H(6S1) 109.3

C(5S)-C(6S)-H(6S2) 109.3

C(1S)-C(6S)-H(6S2) 109.3

H(6S1)-C(6S)-H(6S2) 107.9

C(6S)-C(5S)-C(4S) 111.42(17)

C(6S)-C(5S)-H(5S1) 109.3

C(4S)-C(5S)-H(5S1) 109.3

C(6S)-C(5S)-H(5S2) 109.3

C(4S)-C(5S)-H(5S2) 109.3

H(5S1)-C(5S)-H(5S2) 108.0

_____________________________________________________________

**Figure S12**. The HPLC profiles of separation of (**+**)-evodiakine (**1a**) and (**-**)-evodiakine (**1b**) on chiral IC column (5 *μ*m, 4.6 mm$\times$250 mm), flowrate: 1 mL/min; HPLC: n-hexan/EtOH (0~12min, 90%~10%; 12~16min, 10%; 16~20min, 90%); temperature 25 ºC. (**+**)-evodiakine (**1a**) and (**-**)-evodiakine (**1b**), e.e.% = 0.


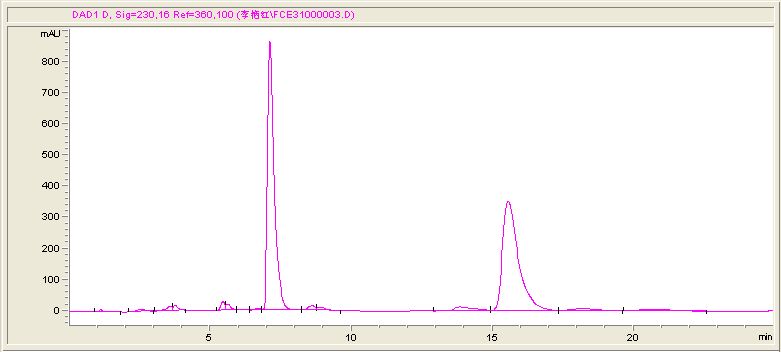

**Figure S13.** ECD spectra of (+)-evodiakine (**1a**) and (-)-evodiakine **(1b**).

The geometry determined from the X-ray analysis was used as the input for the structural optimization by the density functional theory method at the B3LYP/6-31G** level in Gaussian 03 program package,^[1]^ which was further checked by frequency calculation and resulted in no imaginary frequencies. The ECD of the two conformers of **1** (**1a** and **1b**) were then calculated by the TDDFT method at the B3LYP/6-31G** level with the IEPCM model in methanol solution. The calculated ECD curves was generated using SpecDis 1.51^[2]^ with σ=0.16 ev, and UV shifts -2 nm, respectively.


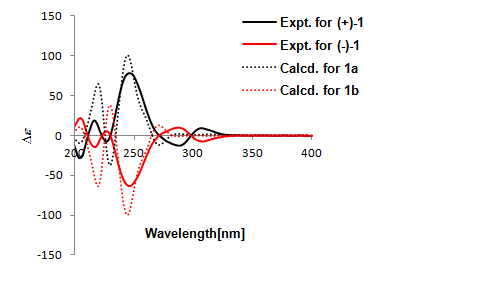


Standard orientation of **1a** at B3LYP/6-31G** level:

---------------------------------------------------------------------

Center Atomic Atomic Coordinates (Angstroms)

Number Number Type X Y Z

---------------------------------------------------------------------

1 8 0 0.291408 2.871517 -0.812538

2 8 0 1.219855 -0.653153 2.927456

3 8 0 -2.260121 2.139341 -0.909355

4 1 0 -1.530061 2.791478 -0.951123

5 7 0 -0.019350 0.367193 1.328621

6 7 0 -0.484627 -0.496067 -0.961331

7 1 0 0.282630 -1.156616 -0.958124

8 7 0 1.953740 1.341742 -0.678231

9 6 0 3.710409 -2.439450 0.083131

10 1 0 4.188765 -3.393274 0.284007

11 6 0 2.738804 -1.948966 0.946950

12 1 0 2.457044 -2.494392 1.841338

13 6 0 2.096403 -0.722571 0.714910

14 6 0 1.081448 -0.323195 1.751573

15 6 0 -0.366998 0.656716 -0.077983

16 6 0 -1.782381 -1.021392 -0.827679

17 6 0 -2.248872 -2.269478 -1.235877

18 1 0 -1.584545 -2.996647 -1.695106

19 6 0 -3.603948 -2.557479 -1.040117

20 1 0 -3.987935 -3.526447 -1.348427

21 6 0 4.064643 -1.689720 -1.040341

22 1 0 4.817187 -2.056600 -1.732611

23 6 0 2.470208 0.041047 -0.414415

24 6 0 2.885311 2.377973 -1.152355

25 1 0 2.918756 2.419692 -2.246952

26 1 0 3.881160 2.162247 -0.763202

27 1 0 2.539872 3.343682 -0.784831

28 6 0 0.634108 1.710998 -0.576836

29 6 0 -1.058835 0.754079 2.299542

30 1 0 -0.591961 1.211395 3.175008

31 1 0 -1.597621 -0.139283 2.637109

32 6 0 -1.950329 1.701481 1.500670

33 1 0 -1.556337 2.723832 1.528436

34 1 0 -2.980678 1.724006 1.865616

35 6 0 -1.869589 1.190751 0.050936

36 6 0 -2.635496 -0.088598 -0.229419

37 6 0 -3.981073 -0.376180 -0.052723

38 1 0 -4.648542 0.356911 0.392855

39 6 0 -4.467560 -1.625958 -0.457098

40 1 0 -5.516405 -1.871026 -0.318456

41 6 0 3.453291 -0.463883 -1.282210

42 1 0 3.733927 0.112272 -2.157412

---------------------------------------------------------------------

Standard orientation of **1b** at B3LYP/6-31G** level:

---------------------------------------------------------------------

Center Atomic Atomic Coordinates (Angstroms)

Number Number Type X Y Z

---------------------------------------------------------------------

1 8 0 -0.291408 2.871517 -0.812538

2 8 0 -1.219855 -0.653153 2.927456

3 8 0 2.260121 2.139341 -0.909355

4 1 0 1.530061 2.791478 -0.951123

5 7 0 0.019350 0.367193 1.328621

6 7 0 0.484627 -0.496067 -0.961331

7 1 0 -0.282630 -1.156616 -0.958124

8 7 0 -1.953740 1.341742 -0.678231

9 6 0 -3.710409 -2.439450 0.083131

10 1 0 -4.188765 -3.393274 0.284007

11 6 0 -2.738804 -1.948966 0.946950

12 1 0 -2.457044 -2.494392 1.841338

13 6 0 -2.096403 -0.722571 0.714910

14 6 0 -1.081448 -0.323195 1.751573

15 6 0 0.366998 0.656716 -0.077983

16 6 0 1.782381 -1.021392 -0.827679

17 6 0 2.248872 -2.269478 -1.235877

18 1 0 1.584545 -2.996647 -1.695106

19 6 0 3.603948 -2.557479 -1.040117

20 1 0 3.987935 -3.526447 -1.348427

21 6 0 -4.064643 -1.689720 -1.040341

22 1 0 -4.817187 -2.056600 -1.732611

23 6 0 -2.470208 0.041047 -0.414415

24 6 0 -2.885311 2.377973 -1.152355

25 1 0 -2.918756 2.419692 -2.246952

26 1 0 -3.881160 2.162247 -0.763202

27 1 0 -2.539872 3.343682 -0.784831

28 6 0 -0.634108 1.710998 -0.576836

29 6 0 1.058835 0.754079 2.299542

30 1 0 0.591961 1.211395 3.175008

31 1 0 1.597621 -0.139283 2.637109

32 6 0 1.950329 1.701481 1.500670

33 1 0 1.556337 2.723832 1.528436

34 1 0 2.980678 1.724006 1.865616

35 6 0 1.869589 1.190751 0.050936

36 6 0 2.635496 -0.088598 -0.229419

37 6 0 3.981073 -0.376180 -0.052723

38 1 0 4.648542 0.356911 0.392855

39 6 0 4.467560 -1.625958 -0.457098

40 1 0 5.516405 -1.871026 -0.318456

41 6 0 -3.453291 -0.463883 -1.282210

42 1 0 -3.733927 0.112272 -2.157412

---------------------------------------------------------------------
